# The Role of the Thioredoxin System in Brain Diseases

**DOI:** 10.3390/antiox11112161

**Published:** 2022-10-31

**Authors:** Geir Bjørklund, Lili Zou, Massimiliano Peana, Christos T. Chasapis, Tony Hangan, Jun Lu, Michael Maes

**Affiliations:** 1 Council for Nutritional and Environmental Medicine, Toften 24, 8610 Mo i Rana, Norway; 2 Hubei Key Laboratory of Tumor Microenvironment and Immunotherapy, College of Basic Medical Sciences, China Three Gorges University, Yichang 443002, China; 3 Department of Chemical, Physical, Mathematical and Natural Sciences, University of Sassari, Via Vienna 2, 07100 Sassari, Italy; 4 Institute of Chemical Biology, National Hellenic Research Foundation, 11635 Athens, Greece; 5 Faculty of Medicine, Ovidius University of Constanta, 900470 Constanta, Romania; 6 School of Pharmaceutical Sciences, Southwest University, Chongqing 400715, China; 7 Department of Psychiatry, Faculty of Medicine, Chulalongkorn University, Pathumwan, Bangkok 10330, Thailand

**Keywords:** antioxidants, oxidative stress, Alzheimer’s disease, Parkinson’s disease, Huntington’s disease, multiple sclerosis, stroke, metals

## Abstract

The thioredoxin system, consisting of thioredoxin (Trx), thioredoxin reductase (TrxR), and NADPH, plays a fundamental role in the control of antioxidant defenses, cell proliferation, redox states, and apoptosis. Aberrations in the Trx system may lead to increased oxidative stress toxicity and neurodegenerative processes. This study reviews the role of the Trx system in the pathophysiology and treatment of Alzheimer’s, Parkinson’s and Huntington’s diseases, brain stroke, and multiple sclerosis. Trx system plays an important role in the pathophysiology of those disorders via multiple interactions through oxidative stress, apoptotic, neuro-immune, and pro-survival pathways. Multiple aberrations in Trx and TrxR systems related to other redox systems and their multiple reciprocal relationships with the neurodegenerative, neuro-inflammatory, and neuro-oxidative pathways are here analyzed. Genetic and environmental factors (nutrition, metals, and toxins) may impact the function of the Trx system, thereby contributing to neuropsychiatric disease. Aberrations in the Trx and TrxR systems could be a promising drug target to prevent and treat neurodegenerative, neuro-inflammatory, neuro-oxidative stress processes, and related brain disorders.

## 1. Introduction

Neurological disorders, including acute stroke and chronic neurodegenerative diseases, present a significant cause of morbidity and mortality worldwide with the extreme medical and financial burden [1,2,3]. Oxidative stress toxicity contributes to the pathophysiology of most neurological diseases, and its magnitude is related to the ability of cellular antioxidants to neutralize the accumulating reactive oxygen species (ROS) [4,5,6,7]. To counteract the detrimental effects of ROS and consequent toxicity and restore the delicate redox hemostasis in the brain, cells are equipped with an endogenous antioxidant defense mechanism consisting of several antioxidant enzymes [8]. The thioredoxin (Trx) system is the major antioxidant system in the brain, and this thiol-dependent redox system comprises Trx, Trx reductase (TrxR), truncated Trx (Trx-80), and NADPH. This system is essential for maintaining the balance of the cellular redox status, and it is involved in regulating redox signaling [9]. Trx is a ubiquitous and multifunctional redox protein, executing its function through its antioxidative, protein-reducing, and signal-transducing activities, and its reduced state is maintained by TrxR [10,11]. Changes in the expression or activities of the Trx antioxidant system have been documented in the brains of patients with neuropsychiatric disorders and in animal models of those disorders [12,13,14,15]. The potential mechanisms of the Trx system components protecting against those diseases not only involve its antioxidant properties but also anti-apoptotic and anti-inflammatory as well as prosurvival pathways [12,13,14,15,16].

The aims of the current study are to review the role of the Trx system in the pathophysiology and treatment of Alzheimer’s disease (AD), age-related decline in memory functions, Parkinson’s disease (PD), Huntington’s disease (HD), brain stroke, and multiple sclerosis (MS) [17,18,19]. We consequently reviewed the relevant pathways, including nitro-oxidative pathways, which play a crucial role in those disorders, the aberrations in the Trx system, and how the Trx system may modulate those pathways, thereby contributing to the pathophysiology of these different brain disorders. We reviewed the many synergistic and antagonistic interactions of the Trx system with the well-established pathways in those disorders and how environmental factors, including metals, dietary deficiencies, pesticides, and infections, may interfere with these interactions. In addition, we reviewed clinical and preclinical studies targeting the Trx system in those disorders to critically evaluate whether the Trx system could be a new drug target to treat these disorders [20].

A state-of-the-art review on the involvement of Trx-related signal pathways in the complex pathophysiology of neurological disorders will provide new insights into the mechanisms of the Trx family proteins in neuropsychiatric disorders, thereby revealing candidate biomarkers and new therapeutic drug targets, which may lead to clinical drug development for the treatment of various neuropsychiatric diseases.

## 2. The Methodology of the Review Analysis

**Figure 1:**
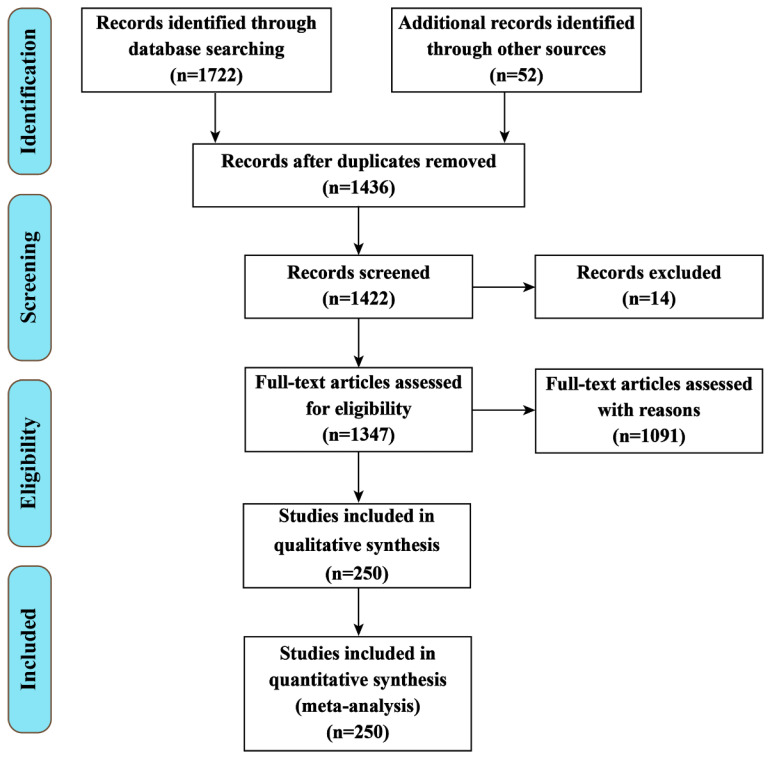
PRISMA 2009 Flow Diagram for the review detailing the database searches, the number of abstracts screened and the full texts retrieved.

The present review was performed according to a systematic search in the databases Science Direct, Web of Science, PubMed, Scopus, and CABI Direct for the years 2000 to 2021 (Figure 1). The keywords used were “brain disease”, “thioredoxin system”, “NADPH”, “thioredoxin reductase”, “thioredoxin” together with one of the following terms: “Alzheimer’s disease”, “Parkinson’s disease”, “Huntington’s disease”, “brain stroke”, or “multiple sclerosis”.

After a first screening, all potentially relevant articles were downloaded from the databases, and the applicable data were extracted and evaluated. Inclusion criteria for this study were: (1) Full-text availability; (2) English language for published full text; (3) Original studies and case reports about brain disease and the Trx system; (4) Research reporting the role of the Trx system, NADPH, TrxR, and Trx in brain disease. Relevant papers were selected and included in this review according to their titles, abstracts, and the mentioned search criteria. Redundant articles or data, conference abstracts, and letters were not evaluated [21].

## 3. The Thioredoxin System

Progressive neuronal dysfunctions may induce neurodegenerative diseases such as AD, PD, and HD. Oxidative damage may frequently occur in the brain because brain tissues consume a high amount of oxygen and contain oxidizable polyunsaturated fatty acids and redox-active metals [22,23,24]. Oxidative stress toxicity plays a critical role in the pathogenesis of several neurodegenerative disorders, especially in elderly individuals [12,25,26]. Moreover, clinical and preclinical studies suggest that altered redox homeostasis increases oxidative stress, as well as decreases levels of antioxidant defenses, may induce activation of stress-related pathways in the brain and peripheral tissues [26,27,28].

The thioredoxin (Trx) system consists of Trx, thioredoxin reductase (TrxR), and NADPH, which are present in various organisms from Archea to humans [29,30]. The Trx system has various functions protecting against oxidative stress and, consequently, serves as a critical antioxidant system in the central nervous system (CNS), DNA synthesis, and apoptosis, thereby maintaining the redox balance in the brain [11,31]. Mammalian cells possess two Trx systems, the cytosolic Trx1 and the mitochondrial Trx2 system. Trx2 has only the two cysteines in its active site, whereas Trx1 has three additional extra cysteines, which play a role in the redox regulation of activity and NO signaling [32]. Trxs, with a dithiol/disulfide active site (-CGPC-), are the main cellular protein disulfide reductases, which act as electron donors for enzymes, including ribonucleotide reductases (RNRs), thioredoxin peroxidases (peroxiredoxins, Prxs), and methionine sulfoxide reductases (MSRs). They play critical roles in pleiotropic cellular effects, such as controlling cell proliferation, redox states, and apoptosis [29,33]. Also, Trx is a serum antioxidant that has been evaluated in the etiology of schizophrenia [34]. Human cytosolic Trx, which is the 12-kDa protein disulfide reductase with the Cys-Gly-Pro-Cys active site and a key component of cellular redox biochemistry and regulation, acts as cocytokine upon leaderless secretion. A 10-kDa C-terminally Trx80 comprising the 80 or 84 N-terminal amino acids is also secreted and present in plasma, where it originally was identified as eosinophilic cytotoxicity enhancing factor [35]. Moreover, TrxRs in higher eukaryotes, including cytosolic TrxR1 and mitochondrial TrxR2, are selenium-dependent dimeric flavoproteins (112–130 kDa). TrxR1 shows a broad substrate specificity that reduces non-disulfide substrates such as hydroperoxides, vitamin C, or selenite. In contrast, TrxRs in bacteria, fungi, and plants are specific dimeric 70 kDa flavoproteins with a redox-active disulfide/dithiol site [29,36]. Three TrxRs are found in mammalian cells, cytosolic TrxR1, mitochondrial TrxR2, and a testis-specific thioredoxin glutathione reductase (TGR) [37]. All mammalian TrxR isozymes are homologous to glutathione reductase (GR) and contain a conserved C-terminal elongation with a cysteine-selenocysteine sequence forming a redox-active selenenyl sulfide/selenol thiol active site.

One of the important endogenous molecules to interact with Trx is thioredoxin interacting protein (TXNIP), which is a negative regulator of Trx function [38]. Cys63 and Cys247 in TXNIP can form the mixed disulfide bond with Trx active site thiols and suppress the activity of Trx and result in oxidative stress [39]. TXNIP located in the nucleus under normal conditions. In response to oxidative stress TXNIP can shuttle into cytosol or mitochondria, which binds and oxidizes Trx1/Trx2, reducing the binding of Trx1/Trx2 with ASK1 and resulting in a ASK1-mediated signaling pathway [40,41]. TXNIP has been found to bind to inflammasome in response to ROS production [42,43]. Trx binds to TXNIP in the steady state. On stimulation of inflammasome activators, ROS will be produced, resulting in the dissociation of TXNIP from Trx and the binding to NLRP3. Then the NLRP3 inflammasome is activated, and the active, mature interleukin 1β (IL-1β) can be produced and secreted after the cleavage from pro-IL-1β precursor by caspase-1 [42]. TXNIP is also shown to be induced by endoplasmic reticulum (ER) stress, to activate IL-1β production via inflammasome, and to mediate ER stress-induced β cell death [44]. In addition, recent study showed that treatment with proteasome inhibitors reversed GAS-induced Txnip degradation. These results indicate that TXNIP may be a potential target against brain disease.

Selenium-based TrxR/Trx and sulfur-based GSH systems are both interactively involved in providing antioxidant activity in cells and tissues, but differences in their relative contributions are evident. Thus, the GSH concentration in neurons is in the range of 0.2 mM [45,46], and this contrasts with the concentrations in hepatocytes, namely around 10 mM [47] or a 50-fold difference. Brain levels of GSH and its precursor cysteine are nearly 10-fold lower than in plasma [48], while selenium content in the brain is only about half of the selenium in plasma [49,50], despite a higher level of aerobic metabolism. Glutathione (GSH) increases the redox impact of neurotrophic factors, which exert epigenetic effects on gene expression via changes in DNA and histone methylation [51]. Accordingly, a normal function of the Trx system is crucial for maintaining brain redox status and for allowing neurotrophic factor signaling to affect gene expression. The latter is particularly crucial during neurodevelopment [52].

Levels of GPx1 and GR in brain mitochondria are low [53], whereas the 2-Cys Prxs, *viz*. Prx1, Prx2, Prx3, Prx4, and Prx5, are highly expressed in the brain and some in brain mitochondria [54,55,56,57,58,59,60,61,62]. This suggests that they may protect mitochondrial DNA in the brain against damage caused by oxidative and nitrative stress and the formation of mutagenic aldehydes as secondary products of lipid peroxidation, such as acrolein [63,64,65] and crotonaldehyde [63,64,65]. 1-Cys Prx (Prx6) is also expressed in the brain [60,66], mainly in glial cells [66,67] and not to any significant extent in neurons [67].

The relative contributions of different scavenging enzymes to H_2_O_2_ removal from isolated mitochondria from brain cells have been measured through in vitro experiments, using polarographic methods for real-time detection of steady-state H_2_O_2_ levels [68]. This research showed that isolated rat brain mitochondria display significant rates of exogenous H_2_O_2_ removal (9–12 nmol/min/mg of protein) in the presence of substrates, indicating a respiration-dependent process [68]. GSH-dependent systems showed only minimal contributions: a 25% decrease in GR inhibition and no effect by GPx inhibition [68]. In contrast, inhibitors of TrxRs, including auranofin, attenuate H_2_O_2_ removal rates in mitochondria by 80% [68]. Furthermore, a 50% decrease in H_2_O_2_ removal was observed following the oxidation of Prx [68]. Decreases in H_2_O_2_ removal are observed in intact dopaminergic neurons with TrxRs inhibition, implicating this mechanism in whole-cell systems [68]. In another study, the specific inhibitors auranofin and 1-chloro-2,4-dinitrobenzene (DNCB) were used to determine the relative contributions of Trx2-dependent and GSH-dependent systems to H_2_O_2_ removal from rat brain mitochondria [69]. The contributions of Trx2-dependent and GSH-dependent systems to ROS detoxification in rat brain mitochondria were 60 ± 20% and 20 ± 15%, respectively [69]. As revealed by aminotriazole inhibition, catalase only contributed to a non-significant extent [69].

## 4. Synergistic and Antagonistic Interactions between Aberrations in Different Signal Transduction Systems

Before discussing the oxidative pathophysiology of the various brain disorders and the involvement of the Trx system in these disorders, we would stress that—as we will show–alterations in the Trx system show many interactions with different signal transduction networks and that combined with these networks the Trx system may induce multiple protective as well as pathophysiological responses. In analogy, synergistic and antagonistic interactions in serotoninergic, dopaminergic, adrenergic, and GABAergic systems may induce more detrimental consequences when all neurotransmitters are simultaneously disturbed. It follows that pharmacological interventions targeting only one of the systems cannot be expected to be effective. In principle, this is similar to other areas of biology, where multiple controls, circular feedback systems, non-linear synergistic cooperation and interdependency of subsystems, and apparent redundancies can be found. Multiple interacting signal pathways, which are present in all nucleated cells of the human body, play a crucial role in controlling cell differentiation and morphogenesis during embryonic development; and multiple genes participate in the control of apparently simple morphological features, including body length [70,71]. There are very fuzzy interactions that are remarkably efficient in the regulatory control of human features and, in fact, quite different from the control systems that engineers design to control chemical processes in chemical factories. However, the reductionist “control engineer” way of thinking is still widespread in medicine—as if the human body were nothing but an expensive new car full of the most recent high-tech gadgets. Therefore, it is important to consider that the associations of the Trx system with the neuropsychiatric disorders discussed here should be interpreted with regard to its many synergistic and antagonistic interactions, feedback, and regulatory effects on oxidative stress toxicity, antioxidant defenses, and apoptotic, immune-inflammatory and prosurvival pathways.

## 5. Alzheimer’s Disease

### 5.1. Pathophysiology of Alzheimer’s Disease

Alzheimer’s disease (AD) is the most critical neurodegenerative disorder of the central nervous system, accounting for more than 60–80% of all dementia cases [17,72] and is a severe healthcare challenge in developed countries in elderly individuals [73,74]. The prevalence estimates indicate that AD affects approximately 15 million persons in the United States [75] and will affect more than 115 million individuals worldwide [76] by the year 2050. According to the Alzheimer’s Association, 13% of older adults over 65 suffer from AD in developed countries [77]. AD progressively affects individuals aged 65 years and older and is the sixth leading cause of early death [78,79]. AD is defined by impairments in memory and executive functions and is accompanied by senile plaques consisting of an accumulation of extracellular amyloid β (Aβ) as well as intracellular neurofibrillary tangles (NFTs) containing a high accumulation of phosphorylated TAU protein, and by loss of neurons, mostly in the hippocampus and cortex [74,79,80,81,82]. Age is the most critical risk factor influencing the heterogeneity and topography of neurodegeneration of Alzheimer’s disease. With a growing elderly population, millions more will be affected by this disease [83,84]. Oxidative stress has been involved in age-related neurodegenerative disorders characterized by progressive neuron dysfunction and cell death. Also, synaptic dysfunctions are involved in AD’s behavioral symptoms and pathophysiology [85] and are strongly associated with oxidative stress toxicity [86]. Additionally, oxidative stress is related to mitochondrial and endoplasmic reticulum dysfunctions, inducing protein misfolding and neuronal apoptosis [74,87].

Various factors may increase the risk of AD, including cardiovascular disease, high blood pressure, diabetes, obesity, high cholesterol, chronic kidney disease, and psychiatric disorders [79,88]. Moreover, familial forms of the disease are linked to genetic mutations in the amyloid precursor protein (APP) gene, presenilin (PS) 1 and 2 genes [89,90], as well as genetic variants in apolipoprotein E allele type 4 (APOE4), which elevates disease risk in most populations [79,91,92,93]. On the other hand, some environmental factors may have a critical role in the pathophysiology and pathogenesis of AD, such as metals, dietary deficiencies, pesticides, and infections [94]. APP is cleaved by γ-secretase and α-secretase to make a soluble, non-amylogenic peptide [95]. However, in AD, APP can be cleaved by γ-secretase and β-secretase to produce the insoluble peptides Aβ40 and Aβ42 as the main shape of amyloid in the cerebral cortex and hippocampus, and while the disease progresses, they spread all over the brain [96]. Also, in AD, neuronal tissue lesions are often associated with the hyperphosphorylation of TAU proteins, producing the NFTs, which aggregate in the neuron’s somatic-dendritic portion [97].

### 5.2. Trx and TrxR in Alzheimer’s Disease

TrxR1 and TrxR2 play critical roles in brain functions. Both TrxRs decrease oxidative stress, neutralize hydrogen peroxide (H_2_O_2_), and regulate redox-sensitive transcription factors that inhibit cellular transcription mechanisms [98]. This protein family may be a protective factor in AD [99]. In brain tissues of AD patients, low levels of Trx1 are observed [97,100] in association with the accumulation of Aβ peptide, and, therefore, it was proposed that Trx1 is a critical protective factor in AD [99]. Decreased expression of Trx1 is observed in both the frontal cortex and the hippocampus CA1 regions of patients suffering from AD [101]. Moreover, treatment with the β-amyloid peptide in human SH-SY5Y neuroblastoma cells may induce early and time-dependent oxidation in Trx1 and Grx1 while Trx1 overexpression effectively preserved cells from Aβ peptide-related toxicity. Trx expression is induced by oxidative stress, thereby regulating redox status and the functions of signaling proteins [102]. Furthermore, TrxR plays a protective role against Aβ toxicity in neuronal cultures and primary hippocampal cultures. Indeed, Trx or TrxR may induce a significant concentration-dependent improvement in cell survival against Aβ-mediated toxicity [99]. Also, administration of reduced Trx to apoptotic cultures of cerebellar granule cells induced a marked reduction in cell death, indicating that a well-functioning Trx/TrxR system improves the survival of cerebellar granule cells [103]. Interestingly, the levels of Trx80 were considerably decreased in AD brain tissue, including in areas with abundant inflammatory changes, indicating that Trx80 failure is a distinct feature of the disease [104]. On the other hand, Trx80, which lacks the C-terminal strand-helix of the Trx fold, induces the proliferation of peripheral blood mononuclear cells [105] and may restrict in vitro accumulation of Aβ as well as Aβ toxicity in SH-SY5Y cells, indicating that increasing Trx1 levels and activities could interact with the toxic effects of Aβ [104]. Moreover, a Tg mouse model overexpressing Aβ showed that TrxR1 activity was significantly decreased at 14 months of age, indicating loss of the Trx1 functions [106]. Trx1 functions in the CNS comprise antioxidant activities and modulate nerve growth factors and other signal transduction pathways [18,107]. Genetic mutations of Trx or TrxR may affect neuronal degeneration [108].

The Prxs system is an antioxidant enzymatic system of emerging significance in the pathophysiology of neuronal aberrations [9]. Gene overexpression, knockdown, and knockout approaches revealed the critical function of Prxs-mediated neuron protection against oxidative stress. Post-translational modifications of neuronal Prxs may cause impairments in their functions as a part of disease pathology. Therefore, members of the Trx superfamily of proteins, the Trx-Prx system, could constitute promising biomarker candidates for the early diagnosis of AD, reflecting their crucial involvement in the treatment and pathogenesis of the disease.

Numerous studies suggest that TrxRs are essential players in antioxidant defenses, cellular redox systems, as well as in growth control, and selenium metabolism [11,33,109]. Decreased selenium is a prominent feature of AD [81,110,111,112]. Selenium (Se), botshowsh as Se-containing compounds and selenoproteins, may be critical in preventing Alzheimer’s pathology [111]. Seleno-L-methionine mixed with vitamin E may have a beneficial role against toxicity from β-amyloid and oxidative stress in cell cultures [113,114]. Also, a mutant APP and presenilin AD rodent model shows the efficacy of organic Se in decreasing AD pathology, reducing the Aβ burden, and minimizing DNA and RNA oxidation [115]. Injections of tricyclodecan-9-ylxanthogenate (D609), a compound that mimics the antioxidant properties of GSH, may decrease Aβ toxicity and oxidative stress by elevating of GPx activity [116].

Furthermore, Trx protein contents are reduced in postmortem AD brains. Still, TrxR activity may be increased [99], which is probably a compensatory mechanism to attenuate activated oxidative stress pathways. Moreover, apoptosis signal-regulating kinase 1 (ASK1) has a vital role in the pathogenesis of neurodegenerative disorders, including AD [117], and Trx is bound directly to the N-terminal region of ASK1 [118] and induces dissociation of Trx from ASK1 in the oxidative stress state. Also, Aβ induces an early, strong, and transient oxidation of glutaredoxin-1 (Grx1) and Trx1.

Mitochondrial dysfunctions coupled with increased oxidative stress (resulting partly from oxidative damage to mitochondrial DNA) may play a significant role in the etiopathogenesis of various degenerative brain diseases, including AD [119,120,121]. Aging mitochondrial DNA [122] will sooner or later lead to enhanced ROS production in mitochondria [123], thereby causing an interaction between mitochondrial DNA aging and Hg inhibition of antioxidant defense enzymes, which will cause enhanced oxidative stress in brain cells. Moreover, the expression of Prx1 is enhanced in an animal model of AD, while this enzyme function is a negative regulator of the expression of the *γ*-secretase complex, which is central to the pathogenesis of AD [124].

### 5.3. Age-Related Decline in Memory Function

There is little doubt that Hg and other Se-antagonistic toxic metals, including Cd and Ag, may cause enhancement of mitochondrial DNA aging in the brain when the exposure is large enough to cause significant inhibition of TrxRs or impaired availability of selenide ions for the synthesis of selenocysteyl-tRNA and incorporation into the Fe-sulphur groups of enzyme complexes in the mitochondrial respiratory chain. Therefore, TrxRs should also be considered one of the prime targets for therapeutic intervention in well-established or suspected Hg poisoning cases.

Enhanced expression of Trx2 and Prx3 (a 2-Cys peroxiredoxin) was observed in the spinal cord and hippocampus of aged dogs [125], suggesting redox regulation of the expression of these proteins with higher intracellular oxidant stress. As described above, oxidative stress occurs in elderly individuals due to mitochondrial DNA aging leading to enhanced production of ROS in the mitochondria [123,126].

Increased oxidative and nitrosative stress in the brain is crucial as a cause of pathological aging processes that ultimately may accumulate in neurodegenerative brain disease. At the same time, the rate-controlling enzymes in the biosynthetic pathways of several important transmitter substances and hormones are inhibited by nitrosative and/or oxidative stress. One example is tryptophan hydroxylase [127,128,129], which is the rate-limiting enzyme in the serotonin biosynthesis pathway, with serotonin and melatonin as end products. Another example is tyrosine hydroxylase [130,131,132], which is a rate-limiting enzyme in the pathway leading to dopamine synthesis, with dopamine and noradrenaline as end products (and for adrenaline in the adrenal glands). A third example is glutamate decarboxylase [133,134,135,136,137], which produces GABA from glutamate, but with one of the glutamate decarboxylase isozymes being much more sensitive to oxidative stress than the other [138].

**Figure 2:**
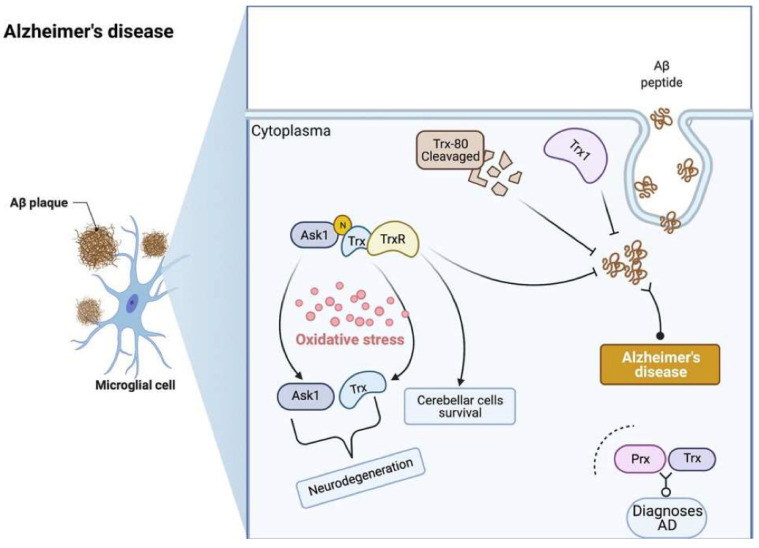
The role of the Trx system in the pathophysiology of Alzheimer’s disease (AD). The combination of Trx with ASK1 may play a role in the neurodegeneration and survival of cerebellar cells. The increased expression of TrxR, Trx1 and Trx80 decreases the accumulation of Aβ in AD patients. Prx and Trx can be used to diagnose AD in lab instead of clinical application. Trx: thioredoxin; TrxR: thioredoxin reductase; Prx: peroxiredoxin; ASK1: apoptosis signal-regulating kinase 1; Aβ: amyloid β.

Figure 2 shows the Trx system’s role in AD pathophysiology. Research has shown that the decreases of Trx1, Trx80, and TrxR in AD brain tissue are distinct disease hallmarks. As a critical protective factor against oxidative stress decreased, Trx and TrxR levels are associated with the accumulation of Aβ and may affect neuronal degeneration. Furthermore, the oxidative state may induce Trx dissociation from ASK1, which, in turn, may induce neuronal death. Also, the Trx-Prx system can be used as a promising biomarker in diagnosing AD.

## 6. Parkinson’s Disease

### 6.1. Pathophysiology of Parkinson’s Disease

Parkinson’s disease (PD) is a common neurodegenerative disorder and the second most prevalent disease, after AD, affecting 0.3% of the general population and 1–3% of the population older than 65 years [139,140,141]. PD is identified by accumulation of α-synuclein within neurons [142], loss of motor control [143], and a progressive, chronic, and profoundoverexpression loss of neuromelanin-containing dopaminergic neurons in the midbrain of the substantia nigra with eosinophilic, intracytoplasmic, proteinaceous inclusion bodies termed Lewy bodies and dystrophic Lewy neurites in surviving neurons [144,145]. Lewy bodies are insoluble proteins, especially α-synuclein, which are produced in these neurons before cell loss [146] as a consequence of incorrect processing of proteins, malfunction of the ubiquitination system, and the unfolded protein response [147]. Most cases of PD are sporadic, although genetic and environmental factors [148] and more likely, a combination of both factors are associated with PD while also familial mutations may be involved [149]. Factors that may induce neurodegenerative processes in the substantia nigra include mitochondrial failure, adenosine triphosphate (ATP) depletion, aberrations in neurotrophic factors, elevated ROS, and excess glutamatergic transmission excitotoxicity, and increased quantities of iron associated with neuromelanin [150].

Some toxins such as 6-hydroxydopamine and 1-methyl-4-phenyl-1,2,3,6-tetrahydropyridine (MPTP) are known to induce dopaminergic neuronal loss in Tg mouse models with null mutations of Parkin, DJ-1, and PINK1 or point mutations of genes located in different PARK loci that carry the same genetic mutations as patients suffering from familial PD [151,152]. Dopamine is used by substantia nigra cells to interact with neurons of the striatum [153] and, therefore, decreased contents of the nigral and, thus, striatal dopamine may cause PD symptoms. A large body of studies showed the effect of oxidative stress in the cascade of events inducing degeneration of dopaminergic neurons in PD [154,155]. Moreover, the oxidative deamination of dopamine and other monoamine transmitters by monoamine oxidase (MAO) is associated with the loss of dopamine neurons in the substantia nigra [156,157].

### 6.2. Trx and TrxR in Parkinson’s Disease

Animal and human MAO-B can produce the neurotoxin 1-methyl-4-phenylpyridinium ion [MPP+] from the MPTP that may induce a severe and irreversible PD-like syndrome [114]. Importantly, Trx1 expression may be decreased by MPP^+^ [158], and its oxidation may induce apoptosis by a caspase-12-dependent pathway and prevent the mitochondrial respiration complex I. Moreover, in vitro and *in vivo*, overexpression of Trx1 has cytoprotective and neuroprotective activities against MPTP [88,159,160]. The most abundant neuronal Prx, Prx2, has an essential role in dopaminergic neuron protection against PD-relevant toxin-induced cell death through modulation of Trx1-ASK-1 interactions and prevents the subsequent stimulation of ASK-1-dependent cell death pathways [161]. Furthermore, neurodegeneration is accompanied by loss of DJ-1, a multifunctional protein with antioxidant activity as well as a transcriptional regulator and stabilizer of nuclear factor erythroid 2-related factor 2 (Nrf-2) [162]. Its oxidative modification has been identified in the brain tissue of PD and AD patients [163]. Moreover, DJ-1, as a redox sensor, can co-immunoprecipitate with ASK-1 during exposure with H_2_O_2_ after dissociation of Trx1 [164,165]. DJ-1 can protect cells against oxidative stress through increased expression of Trx1 via the transcription factor Nrf-2. Therefore, DJ-1 and the Nrf-2-mediated increased Trx1 contents may protect cells against oxidative stress [166]. Furthermore, Mito-TRFS, the first synthetic turn-on probe for mitochondrial TrxR, may provide an accurate discrimination method to image TrxR2 function in living cells [167]. Also, Mito-TRFS showed that PD models are accompanied by severe decrements in TrxR2 activity, suggesting a mechanistic link between dysfunction of TrxR2 and PD etiology.

Selenium may have a critical role in PD by alleviating oxidative stress via selenoproteins [20,112,168]. The plasma levels of selenium may be decreased in PD patients due to higher utilization of selenium for brain selenoprotein production, likely used to attenuate further oxidative damage [169]. A study revealed that blocking the mitochondrial Trx/Prx system induces dopaminergic cells towards mitochondrial dysfunction, elevated steady-state H_2_O_2_, and cell death against toxicants implicated in PD. Also, the lentiviral knockdown of TrxR2 or pharmacological inhibition of TrxR induces dopaminergic cells to sub-toxic concentrations of PD toxicants paraquat (PQ) and 6-hydroxydopamine (6OHDA) [170].

TrxR deficiency may reduce the activity of all 2-Cys Prxs in the brain and may potentiate oxidative stress, mitochondrial dysfunction, and cell death in dopaminergic cells [170]. Therefore, TrxR deficiency is probably associated with a faster progression of PD and should be regarded as a new drug target to treat PD and its progression by enhancing TrxRs and other antioxidant defenses in the brain. Moreover, activated nitro-oxidative stress pathways in PD may also cause comorbid depression, characterized by activated immune and nitro-oxidative stress pathways [171].

Dopamine is an essential neurotransmitter in brain centers, activated during context learning and activities associated with reward and pleasure [172,173,174]. These centers are also activated by nicotine and some psychoactive substances, which may induce dependence, including legal and illegal drugs [175,176,177,178]. Despite having distinct pharmacological targets, these psychoactive agents may enhance dopamine release in the nucleus accumbens [178]. There can be little doubt that inhibition of dopamine synthesis in the brain, accompanied by lowered antioxidant defenses, may increase the likelihood of substituting decreased endogenous dopamine levels with the effects of exogenic sources, including alcohol, smoking, or illegal psychoactive drugs. However, this stimulation induced by drug abuse is much more problematic in intensity and duration than the stimulation induced by natural rewards because exogenic provision tends to affect feedback circuits, reducing endogenic production, producing renewed craving, and thus causing a vicious circle [178].

**Figure 3:**
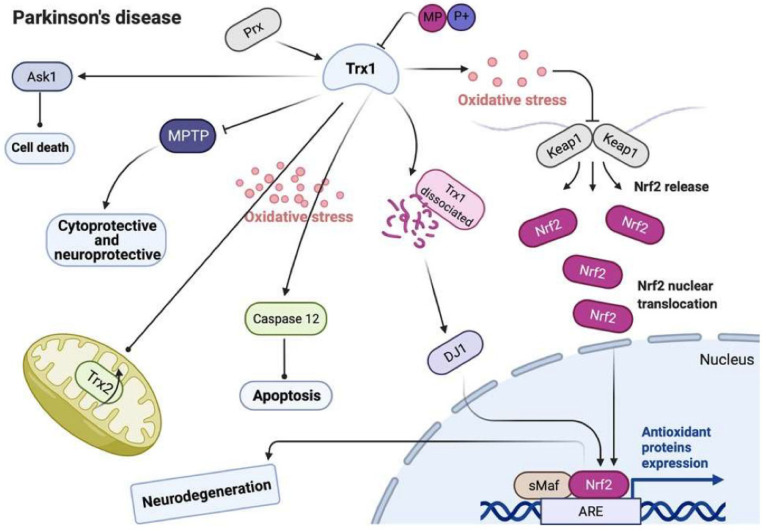
The involvement of Trx1 in pathophysiological signal pathways of Parkinson’s disease (PD). Trx1 participates in a variety of biological pathways of PD. During oxidative conditions, Trx1 stimulates apoptosis signaling caspase 12, which antagonizes the activity of mitochondrial complex I. Trx1 might also suppress MPTP in cytoprotective and neuroprotective mechanisms. When exposed to H_2_O_2_ and dissociated from Trx1, ASK1 can co-immunoprecipitate with DJ-1. Meanwhile, DJ-1 induces expression of Trx1 via transcription factor Nrf-2 to protect cells against oxidative stress. Also, Prx has a protective role via Trx1-ASK1 against dopaminergic neuron cell death. Nrf-2: nuclear factor erythroid 2-related factor 2; MPTP: 1-methyl-4-phenyl-1,2,3,6-tetrahydropyridine; Trx: thioredoxin; TrxR: thioredoxin reductase; Prx: peroxiredoxin; ASK1: apoptosis signal-regulating kinase 1; Aβ: amyloid β.

Figure 3 shows the involvement of Trx1 in the pathophysiological signal pathways in PD. Oxidative stress may induce dissociation of ASK-1 from Trx1 to stimulate ASK-1-dependent cell death, and Prx2 may distinctly protect against the corresponding effect. H_2_O_2_-exposed ASK-1 further co-immunoprecipitates with DJ-1, which mediates an increase of Trx1 against oxidative stress via the Nrf2 signal pathway. Moreover, the decrease and oxidation of Trx1 may induce caspase-12-dependent apoptosis.

## 7. Huntington’s Disease

### 7.1. Pathophysiology of Huntington’s Disease

Huntington’s disease (HD) is a progressive autosomal dominant neurodegenerative disorder [179,180] caused by a trinucleotide CAG repeats in exon-1 of the huntingtin gene (HTT) on the fourth chromosome (4p16.3) [181], resulting in the expression of a polyglutamine-expanded mutant Huntington protein (mHTT) at the amino-terminal end of the protein [182]. The onset of the disease is typically in early to mid-adult life with a range from childhood to advanced age and is defined by an excess of least 39–42 CAG repeats [183,184,185]. The full-length soluble mHTT undergoes enzymatic cleavage to produce soluble N-terminal mHTT polyglutamine involved fragments as monomers, soluble oligomers, and larger insoluble aggregates [186,187]. Soluble N-terminal mHTT fragments are probably the primary drivers of disease progression through accumulation in cells, aberrant interactions with numerous proteins, intracellular inclusion bodies, neuronal death, and possibly direct production of ROS [184,188,189].

The striatum exhibits marked variation in the severity of neurodegeneration involvement [190] with the loss of medium spiny neurons (MSN) in the basal ganglia [181]. In a later phase of the disease, the pathology may spread through the globus pallidus, thalamus, hypothalamus, subthalamic nucleus, substantia nigra, and cerebellum [151,184]. At the onset of disease, the indirect pathway of the basal ganglia could be affected by the inhibitory of dopamine D2-type receptors that extend to the globus pallidus externa (GPe) [172]. In the later stages of the disease, MSNs of the direct excitatory pathway that connects to the globus pallidus internal segment (GPi) are also lost, resulting in symptoms such as akinesia and dystonia [114].

### 7.2. Trx and TrxR in Huntington’s Disease

Trx1 is described as a cytoplasmic and nuclear thiol-disulfide oxidoreductase that has protective effects in acute and chronic models of neurodegeneration. Trx1 transgenic mice display elevated resistance to neuronal degeneration induced by transient focal ischemia [191]. The protective function of Trx1 in various models of neuronal degeneration indicates the critical role of signaling pathways in redox regulation and repair of oxidatively-modified thiols in various proteins.

Nevertheless, oxidative stress in HD is accompanied by elevated GPx levels [192]. The activity of GPx is significantly increased in the brain of HD patients, especially GPx1 in the cerebral cortex and striatum, as well as GPx6 in the striatum. Using quinolinic acid in a rat model of HD, the activity of GPx also increased to induce neurodegeneration [193]. Moreover, GSH and GR act as a backup of human TrxR1 to reduce Trx1, thus preventing cell death caused by aurothioglucose [62]. There is probably a GSH redox cycle dysregulation in HD with a decreased de novo synthesis of GSH in brain cells [194]. This emphasizes the role of a dysfunctional GSH status in HD and targets this system to prevent the onset of HD in relatives of patients genetically predisposed to develop the disease.

GSH is transported by a Na^+^-coupled transport system across the blood-brain barrier in various mammalian species [195,196,197,198,199], including humans [198] with K_M_ values, which are so high [195,200] that they regulate the uptake rate of GSH into the brain. Moreover, Trx1 and the thioredoxin-related transmembrane protein 3 (TMX3) both decrease mHTT in cells, but there is no evidence of direct interaction with mHTT. Moreover, Trx1 and TMX3 decrease striatal neuronal atrophy, suggesting a modulatory role of Trx1 and TMX3 in mouse HD model systems [201]. Moreover, Trx1 is described as a negative regulator of ASK1 and, consequently, is a potential protectant against neurodegeneration [202].

**Figure 4:**
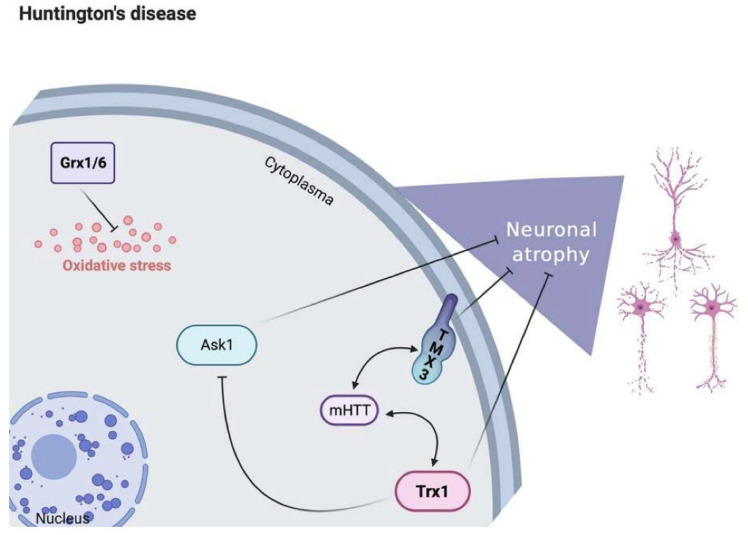
The role of Trx1 and Gpx in the pathophysiology of Huntington’s disease (HD). Trx1 and GPx participate in various biological pathways in relation to HD. The neuron protection activity of Trx1 is achieved via a decrease of both mHTT and ASK1. Moreover, high expression levels of GPx1/6 also play a crucial role against oxidative stress in HD patients. Trx: thioredoxin reductase; TMX3: thioredoxin-related transmembrane protein 3; ASK1: apoptosis signal-regulating kinase 1; GPx: glutathione peroxidase; mHTT: mutant Huntington protein.

Figure 4 shows the role of Trx1 and Gpx in the pathophysiology of HD. Trx1 protects against neuronal atrophy and neurodegeneration, thereby decreasing the expressions of mHTT and ASK1, respectively. Meanwhile, in HD patients, oxidative stress upregulates GPx1 and GPx6.

## 8. Brain Stroke

### 8.1. Pathophysiology of Brain Stroke

Brain stroke induces degeneration of brain tissue that leads to functional impairment with limited brain self-repair [203,204]. Disturbance in the brain blood supply and consequent brain dysfunction is a frequent cause of adult disabilities, which may result in more than half of the patients suffering from long-term disabilities. Brain stroke is the primary cause of adult-acquired disabilities in developing and developed countries, accounting for 9% of deaths worldwide [205,206,207]. The incidence and prevalence of brain stroke are anticipated to increase with the aging of the population [207]. According to the World Health Organization (WHO), annually, 15 million people suffer a stroke worldwide. A brain stroke is an acute-onset clinical syndrome that progresses following a vascular insult to the brain. Following vascular occlusion, a complex chain of molecular events induces irreversible tissue injuries such as failure of energy synthesis, loss of transmembrane ionic gradients dependent on active transport, cell depolarization, and excitotoxicity over the release of excitatory neurotransmitters. Moreover, ROS plays a crucial role in brain injury after ischemic stroke [208,209]. Current data suggest that a rapid increase in ROS production following an acute ischemic stroke due to impaired antioxidant defenses may lead to increased tissue damage [205].

### 8.2. Trx and TrxR in Brain Stroke

Trx has cellular antiapoptotic and anti-inflammatory activities by inhibiting and binding the pro-apoptotic protein ASK1, which stimulates the proapoptotic signaling pathways [202,210]. The Trx1 interacting protein (TXNIP), which may be induced by oxidative stress, is an endogenous inhibitor of Trx1 and is expressed in the brain [1,18,211,212]. Current data suggest that brain redox imbalances are actively involved in ischemic injury [213,214,215]. Furthermore, TXNIP inhibitors induce protection in rodent models of thromboembolic stroke [216] and ischemic-reperfusion injury [212,217]. Trx1 may promote recovery of cognitive functions and neurogenesis following cerebral ischemia in rodent models [218]. In mice, intraperitoneal administration of recombinant human Trx1 may decrease brain damage following ischemic stroke [219]. The neuroprotective activities against the consequences of cerebral ischemia injury may be explained by the anti-oxidative, anti-apoptotic, and anti-inflammatory properties of rhTrx1. Further, it may provide a novel therapeutic approach to decreasing neuronal apoptotic cell death induced by oxidative stress following acute ischemic stroke.

In a rodent stroke model based on middle cerebral artery occlusion injury, inhibition of Trx1 with siRNA induces neuronal apoptosis by stimulating the brain ASK1-JNK/p38 pathway [218,220,221]. Moreover, serum contents of Trx are associated with stroke risk, severity, and lesion volumes, and increased levels may be used as a diagnostic and prognostic biomarker of acute ischemic stroke in a Chinese sample [220,222]. In this respect, serum Trx levels ≥ 20.0 ng/mL are associated with a 9.482-fold increase in the risk of an unfavorable outcome [222].

Also, thioredoxin mimetic (TXM) tetrapeptides, derived from the canonical -CxxC-motif of the Trx1-active site, may reverse oxidative and inflammatory damage mimicking Trx1 activity as well as protect brain cognitive activity after weight-drop closed-head injury in a mouse model of Mild Traumatic Brain Injury (mTBI) [223].

**Figure 5:**
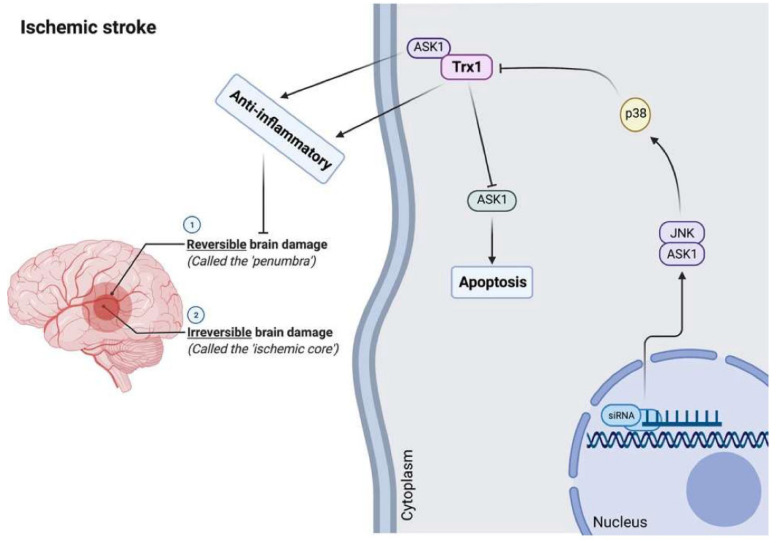
The role of Trx1 in the pathophysiology of ischemic stroke. Trx1 participates in the pathophysiology of brain stroke. Although Trx1 could directly combine with ASK1 to decrease its activity, TXNIP may inhibit Trx1 activity from inducing brain ischemic stroke under oxidative stress conditions. Inhibition of Trx1 with siRNA induces neuronal apoptosis by stimulating the brain ASK1-JNK/p38 signaling pathway.Trx: thioredoxin; TXNIP: Trx1 interacting protein; ASK1: apoptosis signal-regulating kinase 1.

Figure 5 shows the role of Trx1 in the pathophysiology of brain stroke. Although Trx1 may directly inhibit ASK1 and the corresponding ASK1-JNK/P38 pathway leading to anti-apoptosis and anti-inflammation, oxidative stress-induced TXNIP may inhibit its activity inducing stroke, which indicates that Trx1 may protect against ischemic stroke.

## 9. Multiple Sclerosis

### 9.1. Pathophysiology of Multiple Sclerosis

Multiple sclerosis (MS) is a chronic autoimmune and neuroinflammatory disorder characterized by foci of inflammatory demyelination in the optic nerves, spinal cord, and brain, as well as by axonal damage and neuronal degeneration [27,224,225,226]. It is one of the most common neurological disabilities in young adults, with a higher incidence in women. It is accompanied by lowered health-related quality of life, including social withdrawal and unemployment [227,228]. Besides genetic factors, also environmental factors may play a role in MS, including viral infections such as herpesviruses [229] and Human Endogenous Retrovirus (HERVs) [230,231]. Recent studies have demonstrated that oxidative stress plays a crucial role in the pathogenesis of MS. The primary pathologic aberrations in MS comprise axonal and neuronal damage and neuronal loss, leading to permanent neurologic disabilities in MS patients [19,27,232].

Astrocytes are primary regulators of brain oxidative homeostasis, and dysregulation of these cells probably leads to accumulated oxidative damage. On the other hand, the primary regulator of the antioxidant stress defense is Nrf2 [233], as well as peroxisome proliferator-activated receptor gamma coactivator 1-alpha (PGC-1α) [234]. Also, modified redox homeostasis and elevated oxidative stress are primarily a pathway of neurodegeneration and demyelination in the MS brain [235]. Integrated mechanisms regulated by vitagenes operated and encoded by the heat shock proteins (Hsp) Hsp32, Hsp70, Trx, and the sirtuin protein system in the brain are essential for neuronal survival during stressful conditions [27].

### 9.2. Trx and TrxR in Multiple Sclerosis

Numerous enzymes involved in antioxidant defense pathways are upregulated in active MS lesions, including Trx2 [234]. Moreover, in the blood and CSF of MS patients, elevated expression of Trx and sirtuin 1, together with a reduction in the expression of TrxR, were observed [27]. Moreover, Trx has a critical role in the reconstitution of Prx [236], which plays a vital role in preventing oxidative damage in MS lesions and increases astrocyte resilience against oxidative damage [224]. During anti-oxidative stress responses, upregulation of phase II detoxifying enzymes and antioxidant proteins may occur through Nrf2, which has an essential role in inducing the cellular pathways counteracting ROS [237,238]. This Nrf2-induced enzymatic machinery includes enzymes mediating glutathione (GSH) synthesis, the Trx enzyme system, and detoxifying enzymes like heme oxygenases (HO) or NAD(P)H: quinone oxidoreductase 1 (NQO1) [239].

**Figure 6:**
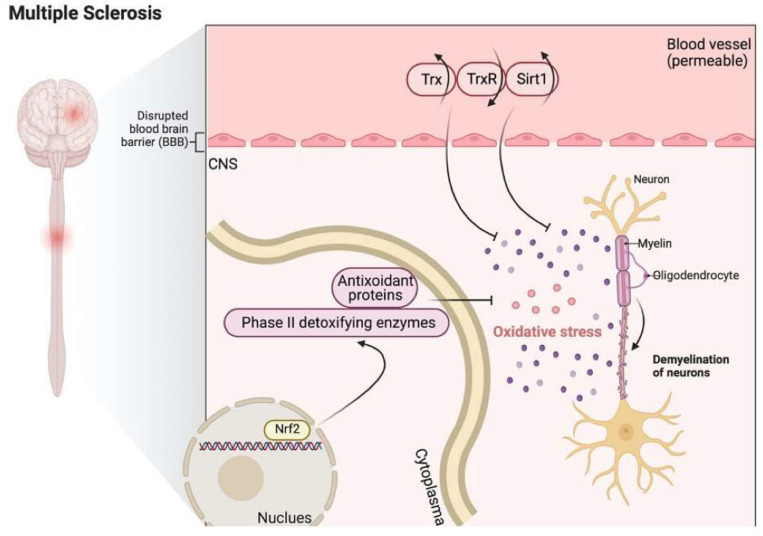
The role of Trx in the pathophysiology of multiple sclerosis (MS). In MS, Trx and sirt1 were upregulated, and TrxR was downregulated. Meanwhile, the activation of the Nrf2 pathway might play a protecting role against oxidative stress by upregulating the expression levels of GSH and Trx. Trx: thioredoxin; TrxR: thioredoxin reductase; Sirt1: sirtuin 1; Nrf2: Nuclear factor (erythroid-derived 2)-like 2.

Figure 6 shows that Trx and sirt1 are upregulated, and TrxR is downregulated in MS. Nrf2-induced GSH synthesis and Trx system activation are involved in anti-oxidative stress responses.

### 9.3. Effects of Metals on the Trx System

Gold (Au) compounds have a long history of use in medicine [240]. Mammalian TrxRs are effectively inhibited by goldthioglucose (aurothioglucose, ATG) and other clinically used drugs [20,241,242,243]. ATG was the first TrxR inhibitor-containing gold [244]. Auranofin is a gold-containing complex used to treat rheumatoid arthritis, which inhibits the activity of TrxR, causing mitochondrial dysfunction, oxidative stress, and mitophagy flux to lysosomes [245,246]. Both ATG and auranofin cannot inhibit GR and GPx, indicating the Sec selenol group of TrxR is the main target. Interestingly, platinum (Pt) and auranofin, which have anti-arthritis effects, may inhibit the selenoprotein TrxR [247]. Also, Palladium (Pd) and Au are remarkably more potent inhibitors of recombinant TrxR1 than Pt. Moreover, gold compounds are highly specific inhibitors of mitochondrial TrxRs, and their activities may induce other functions such as membrane permeability properties. On the other hand, metal ions (cadmium acetate) and metal complexes (cisplatin, zinc pyrithione and tributyltin) significantly inhibit TrxRs activity although with less potency than gold compounds (auranofin, triethylphosphine gold and aurothiomalate, [Au(2,2′-diethylendiamine)Cl]Cl_2_, [(Au(2-(1,1-dimethylbenzyl)-pyridine) (CH_3_COO)_2_], [Au(6-(1,1-dimethylbenzyl)-2,2′-bipyridine)(OH)](PF6), [Au(bipydmb-H)(2,6-xylidine)](PF6)) [20,240,248].

Mercury compounds (methylmercury (MeHg^+^) or inorganic mercury (Hg^2+^) interact with many essential enzymes implicated in antioxidant regulation, including selenoenzymes TrxRs and glutathione peroxidase (GPx) [249,250,251]. Mercury inhibits the Trx system, while the primary molecular mechanism of mercury toxicity is associated with the thiol-selenol in the C-terminal active site of TrxR [252]. Mercury poisoning may cause inhibition of glutamate decarboxylase activity and additionally may reduce dopamine and serotonin levels in the brain [253,254]. There is no doubt that too much Hg may cause harm to the brain [255]. Still, it is a challenging task to assess more quantitatively the consequences of Hg in a health context in comparison with other toxic metals, alcohol, unnatural Ah receptor agonists (including PCBs, PAHs, and various brominated flame retardants) [256] or mutagenic drugs, such as paracetamol [257]. Also, mercury, cadmium, and arsenic exert relatively little GSH oxidation but powerfully cause oxidation of both cytoplasmic Trx1 and mitochondrial Trx2 [258]. The magnitude of mercury, cadmium and arsenic effects was greater for the Trx2 than the Trx1.

As a derivative of Trx, thioredoxin-Albumin fusion (HAS-Trx) counteracted copper-induced neurotoxicity by suppressing ROS production [259]. It is also shown that both iron and copper potentiate GSH loss. Part of the effects of copper on GSH is related to its ability to reduce the activity of glutamate-cysteine ligase [260]. Moreover, Copper, iron, and nickel may induce GSH oxidation, resulting in lower oxidation of cytoplasmic Trx1 and mitochondrial Trx2 [30,242,261].

## 10. Conclusions

There is evidence that Trx and the TrxRs enzyme systems are essential for regulating the cellular redox system, energy metabolism, modulation of immune responses and antioxidant defenses, cell growth and survival, and selenium metabolism in the CNS suppressing neurodegenerative, neuro-oxidative, and neuroinflammatory processes. Many compounds targeting Trx/TrxR functions, such as various flavanoids, gold compounds, platinum compounds, arsenic trioxide, motexafin gadolinium, and nitrous compounds, gained interest in the functions of the Trx/TrxR system. Given the broad range of protective functions, it is evident that Trx and the TrxRs enzyme systems play a crucial role in health and disease, explaining that dysfunctions/overactivation of Trx and TrxRs are associated with various neurodegenerative, neuroinflammatory, or neuro-oxidative brain disorders. The present review indicates multiple aberrations in the Trx and TrxR and other redox systems in AD, PD, HD, stroke, and MS and their multiple reciprocal relationships with the neurodegenerative, neuro-inflammatory, and neuro-oxidative pathways involved in those disorders. Future research should examine whether combining Trx and TrxR and other oxidative stress biomarkers (for example, in machine learning models) could provide novel tool which reflect the severity of neurodegenerative, neuroinflammatory, or neuro-oxidative processes or may be employed as diagnostic or prognostic tools to help in the (differential) diagnosis of AD, PD, HD, stroke, and MS or their staging characteristics. Furthermore, aberrations in the Trx and TrxR systems are probably new drug targets to treat and prevent neurodegenerative, neuroinflammatory, neuro-oxidative stress processes and related brain disorders. Future research should delineate whether these Trx and TrxR targeting drugs may augment the current gold-standard treatment strategies used to treat those disorders.
